# Supplementary figures and images for: BRASS: Permutation methods for binary traits in genetic association studies with structured samples
Source: PLoS Genet. 2023 Nov 7;19(11):e1011020. doi: 10.1371/journal.pgen.1011020 (PMC10656004; doi:10.1371/journal.pgen.1011020)

**S1 Fig. Three-generation pedigree used in the simulation studies.**

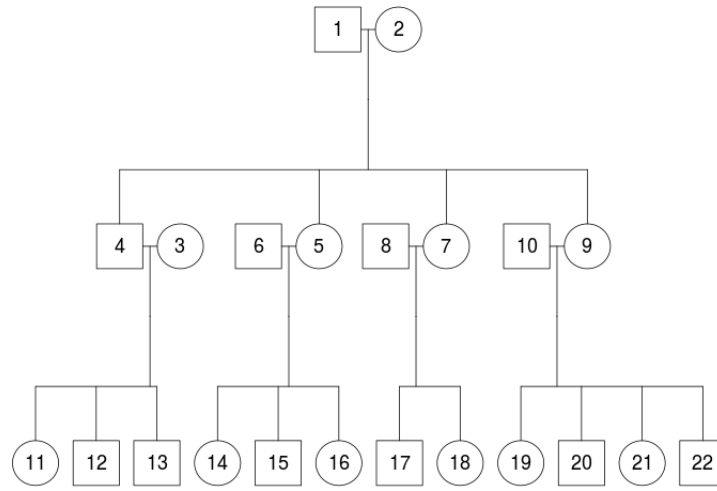

Supplement: S1 Fig — (PDF) [file pgen.1011020.s002.pdf]
