# Supplementary material for: BRASS: Permutation methods for binary traits in genetic association studies with structured samples
Source: PLoS Genet. 2023 Nov 7;19(11):e1011020. doi: 10.1371/journal.pgen.1011020 (PMC10656004; doi:10.1371/journal.pgen.1011020)
Supplement: S2 Fig — (PDF) [file pgen.1011020.s003.pdf]

S2 Fig. Empirical Type 1 Error Rates Omitting a Relevant Covariate at Nominal Level 0.01.

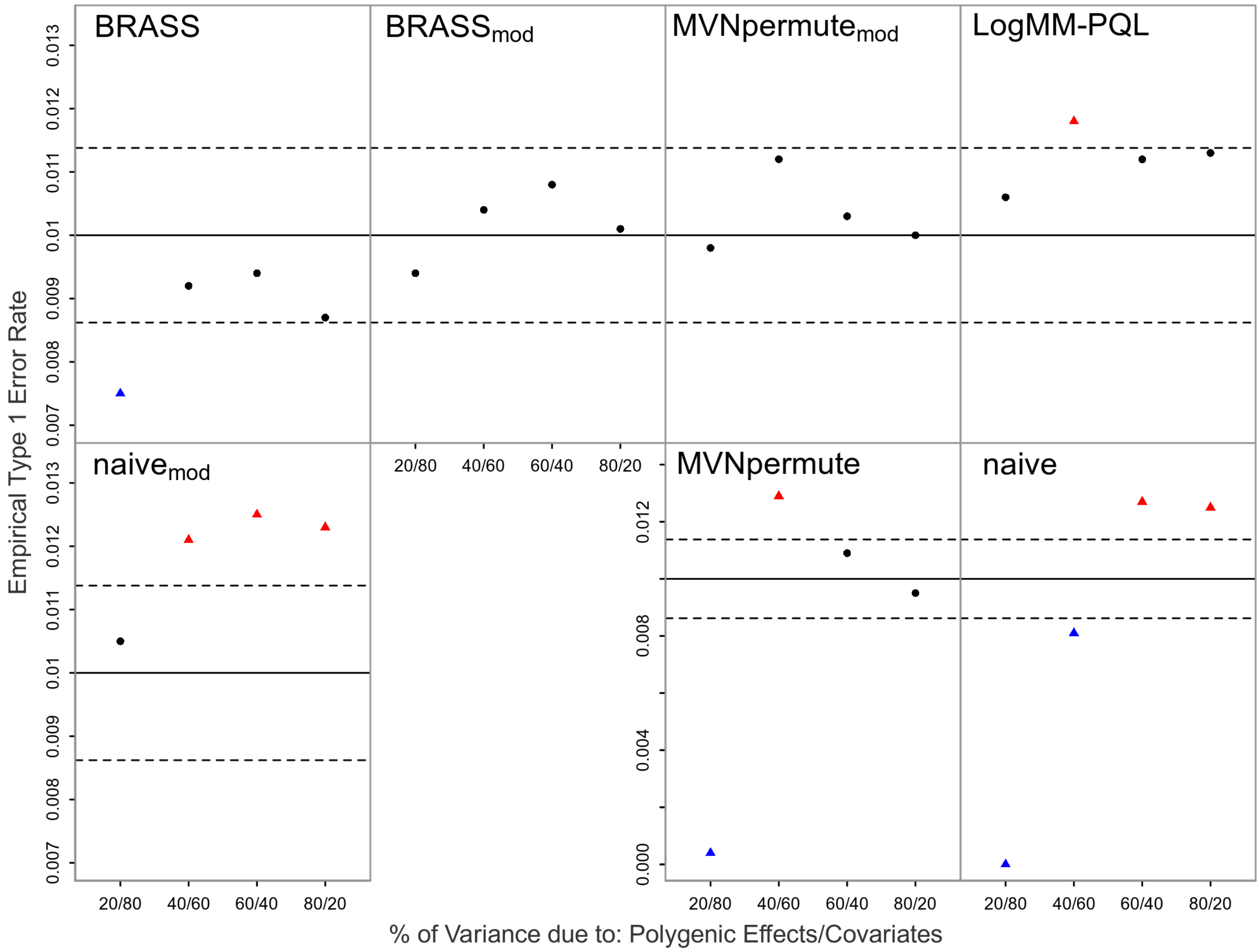

The error rate is based on 20,000 simulated replicates. The solid horizontal line represents the nominal level and the dashed lines represent rejection bounds outside of which the z-test comparing the estimated type 1 error to the nominal level is rejected at level .05. Estimates inside the rejections bounds are represented by circles and those outside the bounds are represented by triangles. Red, black and blue symbols represent inflated, well-controlled and conservative type 1 error rates, respectively. The proportion of variability on the logit scale attributable to polygenic effects vs. covariates is varied from 20 to 80% in increments of 20%. Prevalence is 30%. The effect of the omitted covariate on the trait corresponds to a Wald test p-value of .05 using a linear mixed model.
