# Supplementary material for: BRASS: Permutation methods for binary traits in genetic association studies with structured samples
Source: PLoS Genet. 2023 Nov 7;19(11):e1011020. doi: 10.1371/journal.pgen.1011020 (PMC10656004; doi:10.1371/journal.pgen.1011020)
Supplement: S1 Table — (PDF) [file pgen.1011020.s004.pdf]

S1 Table. Differences between the seven resampling methods compared in the simulation studies.

	**Feature**			
**Method**	Binary trait replicate?	Trait variance is function of mean?	Resampling accounts for correlation in $\mathbf{Y}$?	Resampling accounts for correlation due to parameter estimation?
BRASS	–	$\surd$	$\surd$	$\surd$
BRASS$_{mod}$	$\surd$	$\surd$	$\surd$	$\surd$
LogMM-PQL	$\surd$	$\surd$	$\surd$	–
MVNpermute	–	–	$\surd$	$\surd$
MVNpermute$_{mod}$	$\surd$	–	$\surd$	$\surd$
Naive	–	–	–	–
Naive$_{mod}$	$\surd$	–	–	–

Check marks ($\surd$) indicate that the method incorporates the specified feature.
