# Supplementary material for: BRASS: Permutation methods for binary traits in genetic association studies with structured samples
Source: PLoS Genet. 2023 Nov 7;19(11):e1011020. doi: 10.1371/journal.pgen.1011020 (PMC10656004; doi:10.1371/journal.pgen.1011020)
Supplement: Attachment — Submitted filename: PGENETICS-D-23-00252_reviewer2_Comments to the Authors_May16.pdf [file pgen.1011020.s005.pdf]

Comments to the Authors:

In this paper, the authors have proposed a permutation method called BRASS for binary traits that accommodates familial relatedness in the sample. The authors have demonstrated the robustness of the method to phenotype model misspecification, missing covariates, and case-control ascertainment through comprehensive simulation studies.

Overall, the paper is well-written, and the proposed method appears to be statistically appropriate and practically useful. However, I have a few comments/questions for the authors, mostly regarding the design of the simulation study, with the hope of improving the clarity and consistency of the manuscript.

Main comment:

1. For the simulation study on robustness to ascertainment, the authors have adopted a 1-1 case-control ratio to simulate the data. It would be useful to examine the effect of case-control ascertainment at different case-control ratios for each of the seven resampling methods. Additionally, I suggest that the authors calculate and report the (average) prevalence rate before the case-control ascertainment in this section. To make this simulation study more compelling, the original prevalence rate should ideally be much smaller than the proportion of cases in the case-control sample. It would be also valuable to add a discussion on the effect of extreme case-control imbalance on the method performance; cancer-type of traits in UKB-type of data have this feature.

2. Building upon the previous comment, I suggest that the authors consider conducting a worst-case simulation that simultaneously incorporates case-control ascertainment, missing covariates, and phenotype model misspecification. It would be valuable to determine whether the proposed BRASS and $BRASS_{mod}$ methods still significantly outperform the other methods in this combined setting.

Minor comment:

3. In lines 27-28, the authors stated that "*while an exact permutation test may not be feasible, a permutation-based test that adjusts for the correlation structure is feasible...*". I believe that using the term "approximate permutation test" instead of "permutation-based test" would be more consistent.

4. In lines 34-35, it would be beneficial if the authors provided further clarification on why directly generalizing the MVNpermute method to the generalized linear mixed model (GLMM) would not be feasible for this problem. This additional explanation would enhance the reader's understanding of the methodological constraints and provide a stronger justification for the proposed approach.

5. The authors mentioned an important feature of the proposed BRASS method: its replicates are no longer binary. From my understanding, this feature does not affect the application of the BRASS method in the simulation and real-data example because the association analysis is conducted using a retrospective method called CARAT, rather than the conventional method of logistic regression. However, I suggest providing further rationale on why the non-binary feature of the replicates will not create a problem in the association study. This clarification can be provided after the sentence at lines 288-289 that introduces CARAT. Elaborating on the compatibility of the non-binary replicates with the CARAT methodology would strengthen the justification for using CARAT in the association analysis and alleviate potential concerns about the replicates' non-binary nature.

6. Building upon the previous comment, if the original association analysis is conducted using the conventional logistic regression method, it would be helpful for the authors to provide guidance on whether they would recommend using the modified method $BRASS_{mod}$ instead, despite its potentially lower robustness demonstrated in the simulation studies. This clarification can be included in the discussion section, following the sentence at lines 472-476.

7. At lines 307-309, the authors discussed an adaptive procedure designed to increase the efficiency of the simulation by stopping the permutation process early when a small p-value is obtained. One potential concern with this design is that the empirical type-I-error rate of each method may be biased upward due to the adaptive stopping criterion. To address this concern, I suggest that the authors provide a brief explanation in this paragraph on why such biases is not a significant issue.

8. At line 357, it appears there is a typographical error. It should be "Fig S2" instead of "Fig S1" to accurately reference the figure mentioned.

9. Please clarify the X-axis of Figure 4, as the number of chromosomes are different from the human genome, and presumably only the autosomes were analyzed.

10 . It would be valuable to add some discussions on the treatment of the sex chromosomes, as well as the effect of GxE interaction, which presumably requires some method extension that can be future work.

11. In the application, it is useful to show the performance of the other methods as a) for those scenarios where a method has increased type I error, it would be interesting to see the practical inflation, and b) for those scenarios where a method has deflated type I error, it would be interesting to see if it also identifies the signals. To that end, it would be valuable to state which scenario the application data most likely falls under.

12. The following two pieces of work are potentially relevant:

Zhang and Sun (2023). The hidden factor: accounting for covariate effects in power and sample size computation for a binary trait. *Bioinformatics*: This could be cited on page 4 lines 38-42 to further support the argument that for the binary traits it is important to consider the effect of covariates.

Zhang and Sun (2019). Beyond the traditional simulation design for evaluating type 1 error control: from the 'theoretical' null to 'empirical' null. *Genetic Epidemiology*: This work shows that the size of variance-based tests for continuous traits may depend on how the null data was simulated. As there is a mean and variance relationship for binary traits, it could be valuable to discuss if the proposed method (and the other 7 methods considered) would behave differently between the theoretical and empirical null.
