# Supplementary material for: BRASS: Permutation methods for binary traits in genetic association studies with structured samples
Source: PLoS Genet. 2023 Nov 7;19(11):e1011020. doi: 10.1371/journal.pgen.1011020 (PMC10656004; doi:10.1371/journal.pgen.1011020)
Supplement: Attachment — Submitted filename: Detailed List of Responses.pdf [file pgen.1011020.s006.pdf]

# Detailed List of Responses

## Responses to reviewer 1:

We would like to thank reviewer 1 for taking the time to review the paper and for their thoughtful comments.

1**. The reviewer wrote**: "In the simulation study, the genotype data were generated using 2 sub-population model. How does BRASS perform with more complex population structure, such as more sub-populations and admixture?"

**Our response:**

We first note that our simulation studies already have more complex population structure than just a 2 sub-population model, because we simultaneously include pedigree structure/related individuals as well. Consideration of additional sub-populations and admixture are potentially interesting questions that could be explored with more time and computing resources. However, following the suggestions of the editor, we did not pursue this further for the revision, and instead focused on performing additional simulations that combine ascertainment, imbalanced case-control ratio, missing covariates and phenotype misspecification.

2. **The reviewer wrote**: In the real data analysis with dog GWAS, the original paper described more than two phenotypes. What is the motivation that this study focused on ED and IE? It would be beneficial for readers to see the performance of BRASS across more phenotypes in this dataset. Another option could be the demonstration of BRASS in multiple studies.

**Our response:**

We believe that two data analyses are sufficient for a methodology paper that also includes multiple, massive, simulation studies, especially since the ED and IE data sets we analyzed have extremely different population structure from one another. We have not analyzed any of the other data sets from the Hayward et al. paper besides ED and IE. We chose ED because it had the largest sample size among the case-control data sets, and we chose IE because the results from the previous study seemed potentially interesting but somewhat ambiguous, so we thought it might benefit from a reanalysis.

(Note that aside from the ED data set, which involves multiple breeds, the other case-control phenotypes in the Hayward et al. paper are based on completely separate datasets, consisting of different individuals with different population/breed structure, so analyzing each additional phenotype would involve starting from scratch with quality control, filtering and pruning, assessing and modeling population/breed structure, etc.)

## Responses to reviewer 2:

We would like to thank reviewer 2 for taking the time to review the paper and for their thoughtful comments.

1 & 2. **The reviewer wrote**: "For the simulation study on robustness to ascertainment, the authors have adopted a 1-1 case-control ratio to simulate the data. It would be useful to examine the effect of case-control ascertainment at different case-control ratios for each of the seven resampling methods. Additionally, I suggest that the authors calculate and report the (average) prevalence rate before the case-control ascertainment in this section. To make this simulation study more compelling, the original prevalence rate should ideally be much smaller than the proportion of cases in the case-control sample. It would be also valuable to add a discussion on the effect of extreme case-control imbalance on the method performance; cancer-type of traits in UKB-type of data have this feature.

Building upon the previous comment, I suggest that the authors consider conducting a worst-case simulation that simultaneously incorporates case-control ascertainment, missing covariates, and phenotype model misspecification. It would be valuable to determine whether the proposed BRASS and $BRASS_{mod}$ methods still significantly outperform the other methods in this combined setting."

**Our response:**

For the revision, we have conducted additional simulation studies so that we now consider ascertainment with two different case-control ratios and two different prevalence rates before ascertainment. Furthermore, we have conducted a worst-case simulation study that simultaneously incorporates case-control ascertainment (with imbalanced case-control ratio), missing covariates, and phenotype model misspecification. For each simulation study, the prevalence is calculated and reported (together with the ascertained case-control ratio, if applicable) in the corresponding figure legend.

The results of the 8 new simulation settings are reported in Figs 4 and 5 in the revision. The results turn out to be very similar to those in the original submission, buttressing the original conclusions of the paper that BRASS clearly outperforms the other 6 methods considered.

Aside from the addition of Figs 4 and 5, minor changes to describe the new simulation settings were made at lines 256-257, 265 and 267-270 of the revision (with analogous changes in the S1 Text). A new results subsection, **Robustness to ascertainment, model misspecification and missing covariate, combined,** was added on p. 18 of the revision.

Regarding the reviewer's comment "It would be also valuable to add a discussion on the effect of extreme case-control imbalance on the method performance...":

In the Discussion on lines 542-549 of the revision, we have added:

"Although imbalanced case-control ratio can have an impact on some association testing methods, it would not be expected to have any particular impact on generation of BRASS replicates, because (1) the appropriate modeling of the connection between the mean and the variance for binary data means the method does not break down for small or large probabilities, in contrast to an LMM, and (2) the asymptotic approximations used for inference in logistic

regression are not needed in BRASS. In our simulation results, for the settings with imbalanced case-control ratio (Figs 1, 2, 4, 5), there was no noticeable impact."

3. **The reviewer wrote:** " In lines 27-28, the authors stated that "*while an exact permutation test may not be feasible, a permutation-based test that adjusts for the correlation structure is feasible...*". I believe that using the term "approximate permutation test" instead of "permutation-based test" would be more consistent."

**Our response:**

The reviewer is correct that "approximate" would seem to be an appropriate word to contrast with "exact." However, on reviewing this sentence, we decided to instead simplify it to

"For quantitative traits, a permutation-based test that adjusts for the correlation structure has previously been proposed [9][11]."

4. **The reviewer wrote:** "In lines 34-35, it would be beneficial if the authors provided further clarification on why directly generalizing the MVNpermute method to the generalized linear mixed model (GLMM) would not be feasible for this problem. This additional explanation would enhance the reader's understanding of the methodological constraints and provide a stronger justification for the proposed approach."

**Our response:**

Use of a GLMM would seem to be a natural approach to the problem. In the paper, we actually propose and implement a GLMM approach (logMM-PQL) that fits a GLMM and then uses a parametric bootstrap to generate replicates. In simulations we demonstrate the poor performance of this method.

The reviewer seems to be suggesting some kind of variation on the logMM-PQL approach, though the details of what is being proposed are not completely clear to us. In any case, the first step would presumably be to fit a GLMM to the data, and we explain clearly why this is a problem in lines 157-160 and lines 477-489 in the original manuscript (lines 157-160 and 511-523 in the revision). We did not add anything further related to GLMMs in the revision.

5. **The reviewer wrote**: "The authors mentioned an important feature of the proposed BRASS method: its replicates are no longer binary. From my understanding, this feature does not affect the application of the BRASS method in the simulation and real-data example because the association analysis is conducted using a retrospective method called CARAT, rather than the conventional method of logistic regression. However, I suggest providing further rationale on why the non-binary feature of the replicates will not create a problem in the association study. This clarification can be provided after the sentence at lines 288-289 that introduces CARAT. Elaborating on the compatibility of the non-binary replicates with the CARAT methodology would strengthen the justification for using CARAT in the association analysis and alleviate potential concerns about the replicates' non-binary nature."

**Our response**:

We have done this by inserting the following text at lines 292-301 in the revision:

"One consideration is that only four of the permutation methods we examine create binary replicates, while the other three create non-binary, real-valued replicates. Whether having non-binary replicates is problematic or not depends on the method of analysis. For example, it is not a problem if the data are analyzed using a linear mixed model or using the CARAT [18] or CERAMIC [17] binary trait methods, because these methods are based on solving certain estimating equations and work well provided that the first and second moments of the data are appropriately modeled. However, analysis by ordinary logistic regression would require binary data. To be able to test all 7 methods, we therefore use a (slightly modified) version of CARAT for the stage (3) and (4) association tests, where..."

6. **The reviewer wrote**: "Building upon the previous comment, if the original association analysis is conducted using the conventional logistic regression method, it would be helpful for the authors to provide guidance on whether they would recommend using the modified method $BRASS_{mod}$ instead, despite its potentially lower robustness demonstrated in the simulation studies. This clarification can be included in the discussion section, following the sentence at lines 472-476."

**Our response:**

We have added the following to the Discussion at lines 569-572 of the revision:

"If binary replicates are needed and if the additive polygenic component is estimated to have a low impact on the trait distribution relative to the covariates, then we would recommend either $BRASS_{mod}$ or $MVNpermute_{mod}$, which both provided good type 1 error control in our simulations in those cases."

7. **The reviewer wrote**: "At lines 307-309, the authors discussed an adaptive procedure designed to increase the efficiency of the simulation by stopping the permutation process early when a small p-value is obtained. One potential concern with this design is that the empirical type-I-error rate of each method may be biased upward due to the adaptive stopping criterion. To address this concern, I suggest that the authors provide a brief explanation in this paragraph on why such biases is not a significant issue."

**Our response**:

There was a typo in the S1 Text. In fact, the adaptive procedure stops the permutation process early when a sufficiently **large** p-value is obtained, not a small p-value.

At the place indicated by the reviewer (lines 321-327 in the revision), we have added:

"The amount of downward bias introduced in the empirical type 1 error estimate by this procedure is very small compared to the Monte Carlo sampling variability. For example, when level .01 is tested under the null hypothesis with 20,000 Monte Carlo replicates using the adaptive procedure, the squared bias in the type 1 error estimate due to the adaptive procedure is less than 5e-10, which is several orders of magnitude smaller than the Monte Carlo error variance of 5e-07."

In the S1 Text, on p. 3 in **Adaptive resampling procedure**, we have changed "p < alpha" to "p > alpha" to correct the typo.

8. **The reviewer wrote**: "At line 357, it appears there is a typographical error. It should be "Fig S2" instead of "Fig S1" to accurately reference the figure mentioned. "

**Our response**:

We have corrected this typo.

9. **The reviewer wrote**: "Please clarify the X-axis of Figure 4, as the number of chromosomes are different from the human genome, and presumably only the autosomes were analyzed."

**Our response:**

We have added the following to the legend of old Figure 4 (now Figure 6 in the revision): "SNPs from the 38 autosomes of the domestic dog genome are depicted."

10. **The reviewer wrote**: "It would be valuable to add some discussions on the treatment of the sex chromosomes, as well as the effect of GxE interaction, which presumably requires some method extension that can be future work."

**Our response:**

We have added the following starting at line 538 in the revision:

"Because BRASS generates replicates of the phenotype, it can be used with a variety of genetic predictors. For example, it could be directly applied to association testing of haplotypes, to association testing of variants on sex chromosomes, or to tests of GxE or GxG interaction... In our simulations, we have considered various types of model misspecification and demonstrated robustness of BRASS for association testing. We note that certain applications such as testing of interaction have been shown to be more sensitive to model misspecification than association testing is (Zhang and Sun 2019), so additional sensitivity analyses may be needed when performing interaction analyses, regardless of the method used to assess significance."

11. **The reviwer wrote**: "In the application, it is useful to show the performance of the other methods as a) for those scenarios where a method has increased type I error, it would be interesting to see the practical inflation, and b) for those scenarios where a method has deflated

type I error, it would be interesting to see if it also identifies the signals. To that end, it would be valuable to state which scenario the application data most likely falls under."

**Our response**: We have included the performance results of the other methods in the S1 Text and have added the following sentences to the Results section on lines 434-438:

"The genomewide p-values by the other 6 methods for these same SNPs (detailed in S1 text) are somewhat smaller than those from BRASS, which is in line with the simulation studies showing that the other methods tend to be anti-conservative overall and in particular for models in which polygenic effects are more important than covariates."

12. **The reviewer wrote**: "The following two pieces of work are potentially relevant:

Zhang and Sun (2023). The hidden factor: accounting for covariate effects in power and sample size computation for a binary trait. *Bioinformatics*: This could be cited on page 4 lines 38-42 to further support the argument that for the binary traits it is important to consider the effect of covariates.

Zhang and Sun (2019). Beyond the traditional simulation design for evaluating type 1 error control: from the 'theoretical' null to 'empirical' null. *Genetic Epidemiology*: This work shows that the size of variance-based tests for continuous traits may depend on how the null data was simulated. As there is a mean and variance relationship for binary traits, it could be valuable to discuss if the proposed method (and the other 7 methods considered) would behave differently between the theoretical and empirical null. "

**Our response**:

Zhang and Sun (2023) is now cited in the location suggested by the reviewer (lines 41-42 in the revision) as follows:

"(see, e.g., [12] for importance of modeling covariates in binary data)."

The distinction between "theoretical" and "empirical" null in Zhang and Sun (2019) could be characterized as describing a type of model misspecification in which the assumed model is normal and the true model is a mixture of normals due to an unmodeled covariate. These exact models are not relevant in the binary case, though the general idea of robustness to model misspecification is. The effects of model misspecification are already studied extensively in our simulations (Figs 2, 5, and S2). Zhang and Sun (2019) is now cited in the following two locations:

lines 213-215: "In real data applications, the true trait model is usually not known a priori, and it can be important to assess the impact of model misspecification on type 1 error [25]."

lines 550-554: "We note that certain applications such as testing of interaction have been shown to be more sensitive to model misspecification than association testing is [25], so additional

sensitivity analyses may be needed when performing interaction analyses, regardless of the method used to assess significance."
